# Role of middle managers in dealing with hierarchy and network logics: exploration in the context of Sino-Foreign Cooperative University

**DOI:** 10.3389/fpsyg.2024.1328675

**Published:** 2024-02-16

**Authors:** Jiaxin Li, Xiaojun Zhang

**Affiliations:** ^1^Academy of Future Education, Xi'an Jiaotong-Liverpool University, Suzhou, China; ^2^Department of Sociology, Social Policy and Criminology, University of Liverpool, Liverpool, United Kingdom

**Keywords:** hierarchical organization, institutional complexity, middle managers, network organization, multiple logics

## Abstract

While organizations tend to introduce network mechanism to activate the potential of members in the hierarchical dominated context, it is not clear how individual members deal with the complexity caused by two logics of hierarchy and network. To address this gap, this study focuses on the role of middle managers in collaborating with others in the multiple-logic complexity. We identify three types of collaboration scenarios, top-down, bottom-up, and horizontal, through 27 semi-structured interviews within a Sino-Foreign Cooperative University from 2021 to 2023. Guided by the grounded theory approach, we conceptualize the composite role of middle managers as the translucent hand of explicit and implicit connections, which help us to interpret middle managers' tangibly and intangibly impact under a hybrid organization context. The empirical results also reveal that the boundary perception of authority and responsibility as an important factor determines middle managers' awareness of power involvement in cooperation. The findings extend the understanding of middle managers in network organizations in the higher education context and provide suggestions for the dynamic role of middle managers and hybrid university management in the information age.

## 1 Introduction

Organizational structure is “the formal allocation of work roles and administrative mechanisms to control and integrate work activities” (Robbins, 1990). In the higher education context, most universities adopt a hierarchical structure under which the university is divided into different schools and departments, with each school focusing on a particular discipline. Individual cooperation within schools and departments is maintained through formal power relations (Hellawell and Hancock, 2001). Although the hierarchical structure has supported universities' operations for centuries, the emerging knowledge economy and disruptive information technologies challenge its effectiveness in the information age. The traditional hierarchical organizational structure, which relies on a linear and top-down power chain (Diefenbach and Sillince, 2011; Monteiro and Adler, 2022), limits the cross-departmental cooperation. Therefore, how universities can respond to the educational needs of an emerging information society and change their structure to support educational development has become a considerable challenge.

The network organizational structure was proposed to address this challenge (Powell, 1990; Miles and Snow, 1992; Moretti, 2017; Brennecke, 2020), and as a response of the twenty-first-century organizations to societal needs and changing environment (Drucker, 2007; Aita, 2017). Network organizations are described as voluntary, delayered cooperative networks that can improve the flexibility and adaptability of organizations (Alvarez and Ferreira, 1995; Jacobsen et al., 2022). Moreover, network organization is viewed as a counter model to hierarchical organization, and the most apparent difference between them is the internal cooperation pattern (Powell, 1990; Aita, 2017). Network organization emphasizes horizontal cooperation while hierarchical organization emphasizes pyramid cooperation.

The network organizational structure can flexibly construct a unique set of internal and external linkages for each unique project (Baker, 1992; Amati et al., 2021). Network organizations can thus be divided into intra- and inter-network organizations. Here, we focus on intra-network cooperation, which is described as “a dynamic and strategically planned network of self-programmed, self-directed units based on decentralization, participation, and coordination” (Castells, 2012; Jacobsen et al., 2022). Moreover, intra-network cooperation is based on shared responsibility among colleagues and not on a superior-subordinate relationship (Miles and Snow, 1992; Moretti, 2017).

**Figure 1:**
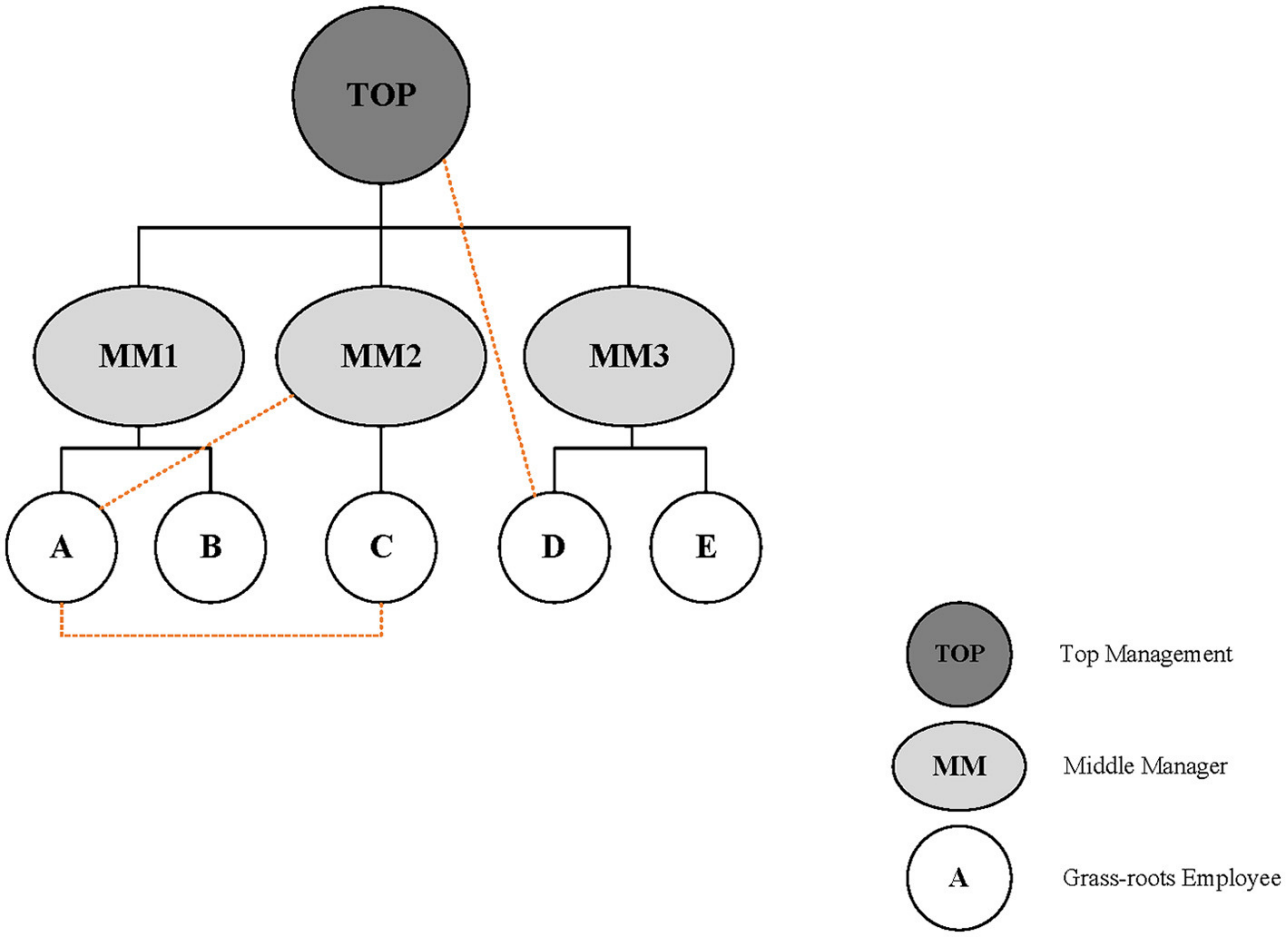
The way of network cooperation.

Figure 1 shows the reflection of intra-network cooperation in a university, that is, (1) employee A can directly cooperate with employee C without the need for permission from middle managers; (2) employee A can be flexibly involved in the project of a middle manager; and (3) employee C can directly contact the top management for cooperation. The three types of flexible cooperation among employees are shown in Figure 1, which indicates the key feature of intra-network cooperation: it breaks through the logic of power, and such cooperation are task-oriented rather than position-oriented.

However, there are still some challenges with the intra-network organizational structure as the role of members in network organizations becomes increasingly complex in the ever-changing environment. Therefore, exploring how employees from different departments within an organization work together helps identify cooperation patterns within an organization. The identity of employees in a network organization focuses on role in the project rather than fixed position in a hierarchical organization (Aita, 2017). Moreover, middle managers are unique members of organizations. They are regarded as intermediate transtainers who supervise grassroots employees and stand between them and the top level of the organization (Sondak, 2005; Tarakci et al., 2018). The role of middle managers in cooperation has two effects. First, middle managers can help delineate the boundaries of staff responsibilities in their daily work. Second, intangible behavioral encouragement from middle managers also drives employee collaboration, which breaks through the logic of power. Therefore, middle managers can be a special viewpoint for exploring intra-network cooperation in organizations.

Middle managers play a fixed role in a hierarchical context. When the organization starts transforming to the network organizational structure, middle managers must face the challenge of choice. Delayering the structure will shake the middle managers' positions. Some scholars argue that the excessive freedom of network organizations can lead to organizational inertia (Miles and Snow, 1992; Tunisini and Marchiori, 2020). In the complex and changing organizational context, the role of middle managers is still ambiguous. There is a lack of research on how middle managers make choices when hierarchy and network coexist. It remains unclear whether middle managers will be diminished in influence during the organizational change or middle managers will become necessary for moderate management intervention when hierarchy and network coexist. Therefore, this study applies a lens of middle managers' role to explore cooperation patterns in the intra-network organization from the university perspective. This study was guided by two research questions:

1. What is the role of middle managers in cooperating under the hybrid organization context that hierarchy and network coexist?
2. What factor determines the middle managers' choice in adopting hierarchical or network cooperation?

## 2 Theoretical background

### 2.1 Hierarchical and network organizations

Various organizations have adopted hierarchical organizational structure for long periods to cope with the need for stable management and resource integration. With a stable management pyramid, each manager manages a certain number of people, cascading upward to achieve centralization. Hierarchical organizations have considerably contributed to increasing labor productivity. Moreover, it adopts a line functional organizational structure, achieving centralization of power through line management based on the line of command-and-control (Diefenbach and Sillince, 2011). Therefore, cooperation within hierarchical organizations is often based on power relations. That is, the subordinates follow the orders and arrangements of their superiors. The power chain is vertical rather than horizontal. A hierarchical structure refers to clear departmental boundaries, clean lines of authority, detailed reporting mechanisms, and formal decision-making procedures that are particularly well-suited for mass production and distribution (Powell, 1990; Reitzig and Maciejovsky, 2015).

However, hierarchical reporting under a pyramidal power structure hinders cooperation among members and cross-departmental collaboration (Bleiklie et al., 2015). Particularly, when hierarchical forms face sharp fluctuations in demand and unexpected changes, members' liabilities are suspended (Powell, 1990). It also reflects the constraints on the speed of hierarchical organization when reacting to environmental changes. With the rapid development and wide application of Information and Communication Technology (ICT), the drawbacks of traditional hierarchical organizational structure have been exposed (Sobolewska and Kisielnicki, 2021). Universities are supposed to create new learning opportunities and encourage educational innovations to transform learning and teaching processes by integrating educational technologies (Li et al., 2022). Malik (2023) also empathizes with the innovation of the Sino-Foreign Cooperative University (SFCU) in the education system through the technical core and institutional conformance. In terms of SFCU, it is different from conventional universities because of its multicultural backgrounds and governance, which bring challenges for organizational management.

Additionally, there is a growing need for interdisciplinary and cross-departmental cooperation among universities. Nevertheless, it is challenging to realize this innovation through the compulsory deployment of power. The core resources of universities are distributed to each employee so that educational innovations are inevitably linked to the knowledge and competence of each academic staff. Innovative knowledge organizations and employees prefer a relaxed, self-directed working environment. In that sense, traditional hierarchical structures are too cumbersome to effectively support innovation in universities. Hence, to keep pace with the rapid educational development, SFCUs need to reconsider the organizational structure to integrate dispersed knowledge resources better for educational innovations.

The universality and functionality of organizational forms cannot simply be classified as a market or hierarchy (Podolny and Page, 1998; Moretti, 2017). Organizations respond to an increasingly competitive global business environment by moving away from centrally coordinated, multilevel hierarchies and toward more flexible structures that closely resemble networks rather than traditional pyramids (Kotter, 2012). In the era of volatility, uncertainty, complexity, and ambiguity (VUCA), a new and flexible organizational structure needs to be applied to cope with the ever-changing external environment. For instance, Internet companies that still adopt a hierarchical organizational structure may delay information processing and resource sharing. In summary, the shortcomings of control-centric hierarchical organizations have gradually been exposed.

Therefore, many organizations are beginning to explore flat management that addresses flexible teamwork. It includes reducing management layers, controlling management scope, and improving organizational efficiency (Walton, 1985; West, 2012). Simultaneously, it accelerates information flow and enhances organizations' decision-making efficiency, which can help promptly respond to changes in the internal and external environment with agility and flexibility (Vega-Redondo, 2013). Therefore, the emergence of ICT-based network organizations compensates for defects in hierarchical organizations. Consequently, network organizations can also be treated as a trend in organizational structure development.

The shift to delayering organizational structure and cross-functional teams requires empowered decision-making (Miles and Snow, 1992; Serrat, 2017). In network structures, authority, typically held by individuals or groups with critical skills, is decentralized to perform specific tasks or functions (Alvarez and Ferreira, 1995). Communication flows are free, and there is shared access to information and knowledge in a network organization (Rodan and Galunic, 2004). Organization members handle organizational affairs together with the support of “sharing” and “coordination” goals; loose and flexible organizational culture concepts are also helpful to maintain the organizational operation and realize organizational cooperation (Tsai, 2002). A network organization is a value-oriented, vision-driven ecosystem that depends on the operation of the network-based support platform, which aims to realize the value-added, co-existence, and win-win situation of participants on the platform (Kotter, 2014).

The basics of network organization rise from the “network” theory (Borgatti and Halgin, 2011; Serrat, 2017). Baker (1992) remarked that network organizations can flexibly construct different internal and external linkages for each unique project (Ekbia and Kling, 2005). From this perspective, network organizations can be divided into inter- and intra-network organizations. The former refers to inter-organizational alliances, which explore the cooperation mechanism between independent organizations and can be described as external networks (Alvarez and Ferreira, 1995). In comparison, an intra-network organization relies more on a flexible link that focuses on flattening and cross-functional integration from inside one organization, which can be seen as an internal organizational structure (Flap et al., 1998; Casciaro and Lobo, 2015). Moreover, intra-network organizations can improve company performance and efficiency (Snow et al., 1992; Kisielnicki and Sobolewska, 2019; Brennecke, 2020). Network organizations can accelerate information flow and enhance the decision-making efficiency, which can help companies respond to changes in the external market environment (Baker, 1992; Miles and Snow, 1992; Ekbia and Kling, 2005). For example, Powell (1990) stated that networks possess the comparative advantage in coping with an environment that places a premium on innovation and customized products. Podolny and Page (1998) further proposed that network forms of organizations foster learning, provide a variety of economic benefits, facilitate the management of resource dependencies, and provide considerable autonomy for employees.

While previous studies analyzed the starting point for creating effective profits, limited attention has been paid to intra-network organizations among non-profit organizations. A university is a knowledge-based organization with a vision of knowledge creation and a mission of talent cultivation. Given the organizational changes brought about by the digital wave, it is worth exploring how intra-network organizations promote interdisciplinary knowledge creation. Knowledge creation cannot be achieved without the trust and collaboration among different members. Therefore, it is essential to explore intra-network cooperation patterns from the perspective of member cooperation. Members are an integral part of the organizational structure. In particular, middle managers are special members holding a bridging position with the challenge of an unpredictable existence in organizational structure changes. Hence, it is worthwhile to explore what changes in the role of middle managers have led to what kind of changes in the university structure. This could reveal the nature of member cooperation within a university organization.

### 2.2 Role of middle managers

#### 2.2.1 Middle managers in hierarchical cooperation

The Heads of Department (HoDs) are mainly involved in university management and commonly regarded as middle managers (Hellawell and Hancock, 2001; Kallenberg, 2015). Under the traditional hierarchical university management, HoDs are the academic leaders of the discipline and responsible for teaching and research arrangement (Ghavifekr and Ibrahim, 2014). However, HoDs also take the role of administrative managers who hold a bridging position between grassroots staff and top university management (Clegg and McAuley, 2005). Therefore, HoDs' role is influenced by a combination of academic and administrative powers. In summary, as middle managers, HoDs hold a particular identity with different roles, so their importance is self-evident.

Middle managers' role in hierarchical university organizations can generally be summarized into two categories: structural and functional (Table 1). The structural role is commonly described as a link, linking pins, and bridging positions (Hellawell and Hancock, 2001; Kallenberg, 2015; Tarakci et al., 2018). They are managers with the power to make decisions. They are also executives who must complete tasks arranged by the top managers. Additionally, they act as a communication bridge that accepts top managers' instructions and coordinates them with their subordinates. Regarding their functional role, some scholars have adopted implementers, facilitators, coordinators, and networks to describe the multiple functions of middle managers in improving organizational performance and innovation (Kallenberg, 2015; Leithwood, 2016; Saibene et al., 2020). As implementers, the HoDs take instructions from superiors to refine and implement their general objectives (Loh and Hu, 2021). Facilitators identify the potential opportunities for collaboration and promote innovative activities within departments. Coordinators are responsible for the departmental asset operations and resource allocation. Networkers establish relationships with various departments, such as service centers for access to funds and equipment and the academic affairs department for policy support.

**Table 1:** Role classification of middle managers in hierarchical organization.

Structural	Link
	Linking pins
	Bridging position
Functional	Implementor
	Facilitator
	Coordinator
	Networker

In summary, HoDs as middle managers play a pivotal role in hierarchical organizations. However, organizations are transiting to networks, which reduces hierarchical transmissions and mainly affects the middle management. Therefore, the survival space for middle managers was sharply compressed. Simultaneously, limited growth and competition have jointly led to professional crisis and the unpredictable existence of middle managers (Rouleau and Balogun, 2011). This illustrates a new challenge for the middle managers' traditional roles.

#### 2.2.2 Middle managers in network cooperation

Network organizations provide a convenient platform for flexible cooperation among employees and promote information flow (Snow et al., 1992; Jacobsen et al., 2022). Network organization studies consider network cooperation to break through the logic of power (Powell, 1990; Sandström and Carlsson, 2008; Massa and O'Mahony, 2021). However, when the flexible cooperation of grassroots breaks through the power control of the middle (as illustrated in Figure 1), the traditional role of middle managers will face an unprecedented challenge. There is still a lack of attention to network cooperation from the perspective of middle managers in the universities. To address this research gap, we discuss the controversial role of middle managers in universities by exploring a new method of network cooperation as illustrated in Figure 1. Additionally, the factors that influences middle-level involvement in hierarchical or network cooperation remains unclear. Hence, we investigate the factor influencing middle managers' role choice through an integrated view of hierarchy and network.

## 3 Method

In this qualitative study, the grounded theory method was adopted to capture the complexities of this phenomenon as it involves an ongoing inductive analysis of primary sources and empirical facts. The research questions were originated from respondents and generated from data; concepts were summarized from the original data, and theories were constructed using empirical data (Charmaz, 2014). To investigate cooperation patterns by analyzing middle managers' role in a network organization in the university context, an in-depth exploration of organizational structure change was conducted with Y University, which is a typical Sino-Foreign Cooperative University. Y University initially established a network supporting platform to reduce administrative barriers of hierarchy. Based on this platform, Y University attempts to strengthen the links between different disciplines to develop student-centered knowledge activities for effective learning and integrated cultivation. Neither the senior management team nor the administration directly interferes with the development of talent cultivation and research activities. Y University's structure aims to create a service-oriented support system rather than cumbersome management. For example, the school structure is divided into the Dean, Head of the department, and academic staff. The department is the primary teaching organization unit. Moreover, Y University integrated academic departments, research institutes, research centers, and language centers that promote flexible cross-departmental and cross-discipline collaboration for student cultivation.

## 4 Data collection and tool

Data collection was approved by the Research Ethics Committee. Primary data was mainly collected through in-depth and semi-structured interviews from 2021 to 2023. In total, a diverse range of university members comprising 27 staffs (i.e., head of the department, head of the center, grass-roots employee) from various departments of Y University participated in the interviews. Since Y University is one of the SFCUs, foreign employees occupy a certain proportion. Hence, the local and foreign employees were considered during the interview sampling.

Two rounds of face-to-face or online interviews were conducted, and the duration of each interview was around 40–90 min. In addition to the open-ended questions, some follow-up questions were asked according to the interviewee's performance. Questions for general academic staff included “Which departments do you regularly work with?” “Why do you work with these departments so often? And especially work with whom?” “Who leads the cooperation process?” and “Who usually make the decisions or propose the plan in the cooperation?” Questions for HoD included “Who usually initiates the cooperation?” “What difficulties have been encountered in the cooperation?” “Who propose the solutions to these difficulties?” and “How do you see yourself involved in the cooperation?” Besides, all interviews were recorded and transcribed. Note-taking was done during the interview process for the reflection. Besides primary data, the secondary data collection consists of two types of archival documents. Two publicity books and 31 media coverage were chosen to allow a deeper insight into the university development, organizational governance, and some main activities of Y University.

## 5 Data analysis

Based on the data gathering, the grounded theory method was employed to analyze the patterns of cooperation through which we further explored the role of middle managers (HoDs) in the cooperation. This study follows the process of initial coding, focused coding, and theoretical coding (Charmaz, 2014). Initial codes are “provisional, comparative, and grounded in the data,” leading us to subsequent conclusions about establishing our fundamental conceptual categories (Charmaz, 2014). After that, the focused coding tried to make initial codes more directed, selective, and conceptual (Thornberg and Charmaz, 2014). Theoretical codes are integrative and lend form to the focused codes which help the researchers tell an analytical story with coherence and move it in a theoretical direction (Glaser, 1978; Charmaz, 2014).

**Figure 2:**
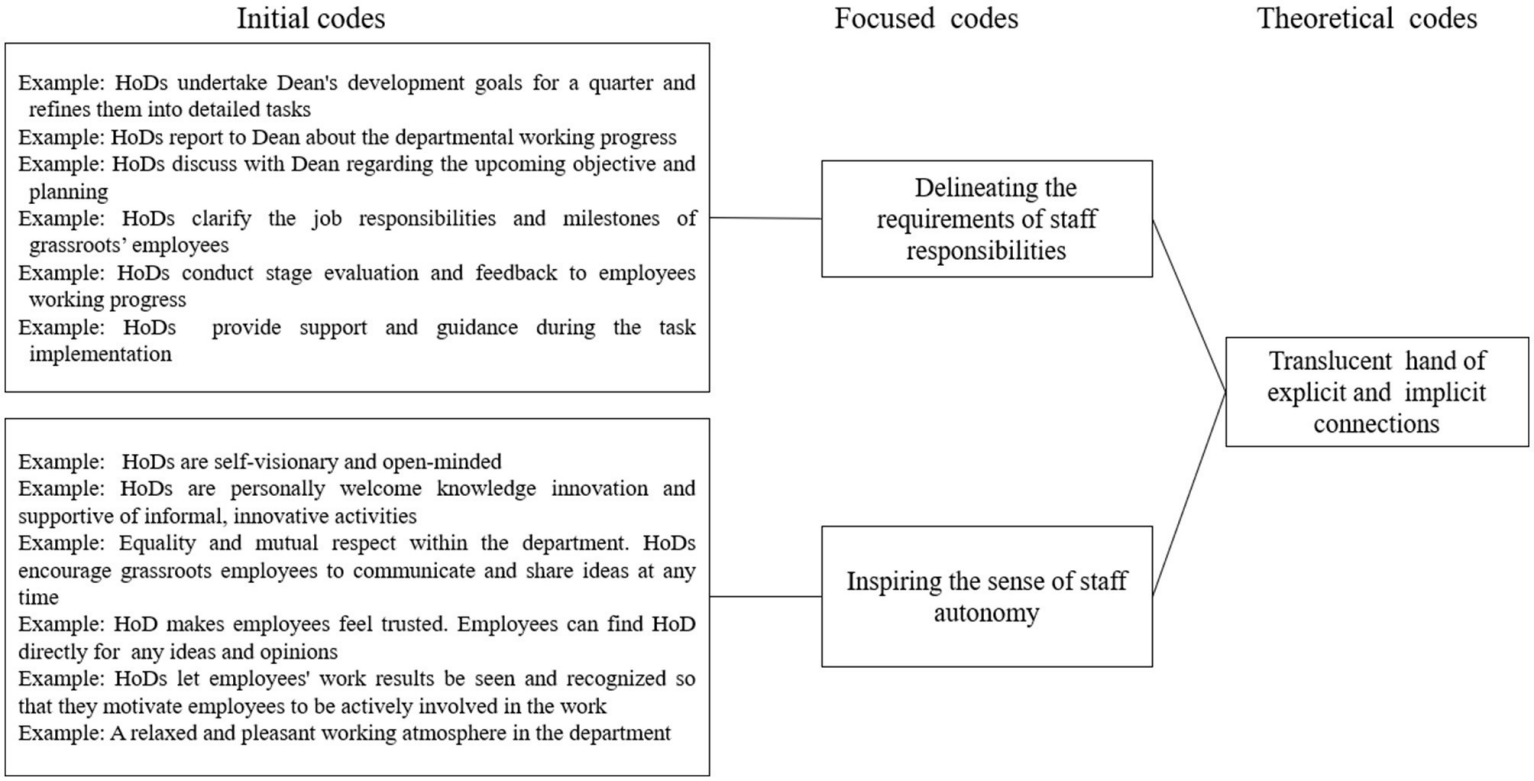
The data-analysis structure for RQ1.

Hence, following the guidance of grounded theory approach, the incident content was coded word-by-word and line-by-line, which explored the role of middle managers (HoDs) in different cooperation. Twelve initial codes helped separate the data into categories and demonstrated HoDs' role during the cooperation process. Two focused codes (delineating the boundaries of staff responsibilities; behavioral encouragement for employee collaboration advancement) were shifted and conceptualized from the initial codes to make them more directed and selective. Finally, a theoretical code was developed to describe the role of HoDs, which is the translucent hand of explicit and implicit connections to answer the RQ1 (Figure 2).

**Figure 3:**
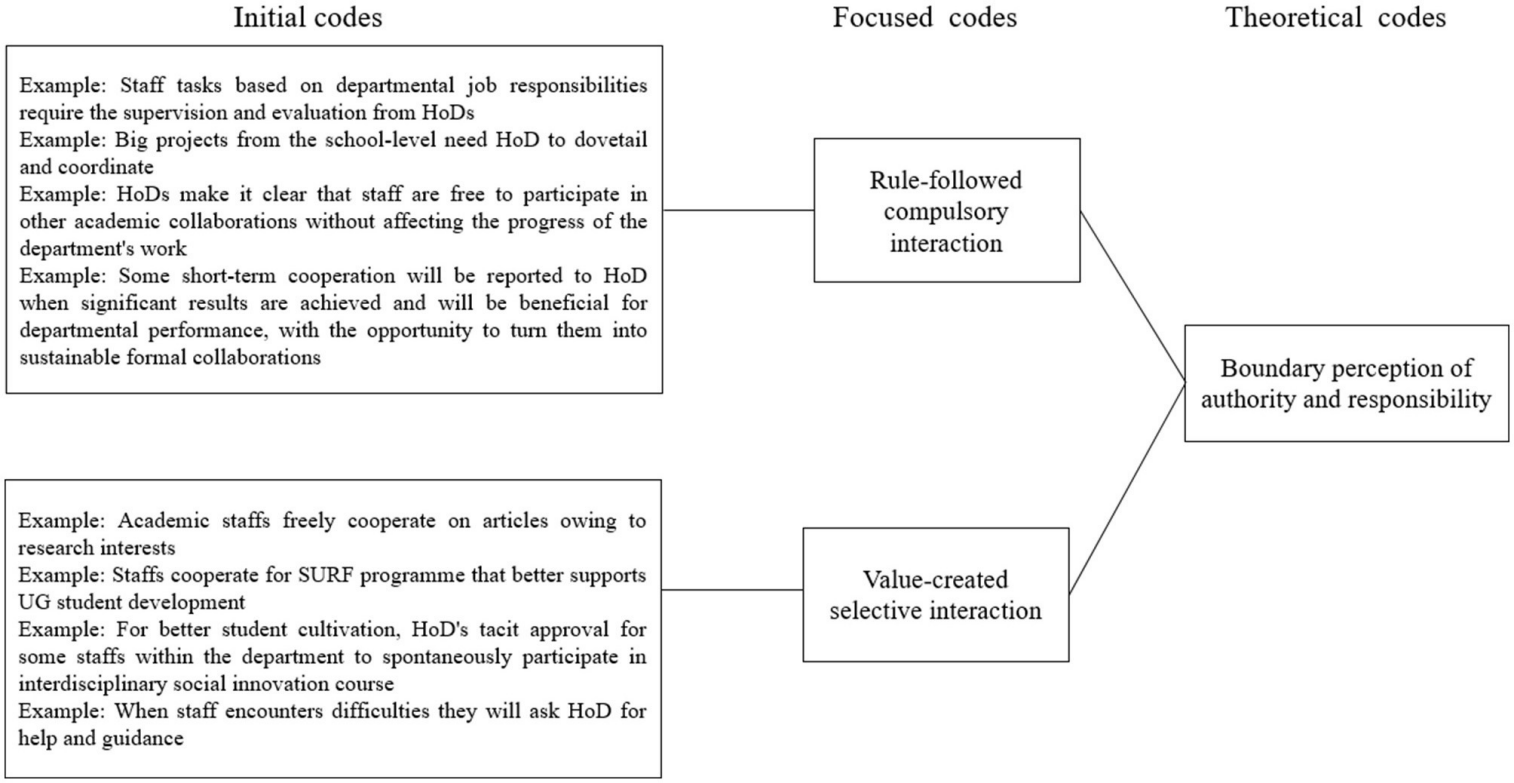
The data-analysis structure for RQ2.

Moreover, two focused codes (rule-followed compulsory interaction and value-created selective interaction) were developed based on the nine initial codes to further analyze the factors affecting middle managers' role in different types of interaction with employees (Figure 3). Finally, we arrived at one theoretical code to answer what factor determines middle managers' choice in adopting hierarchical or network cooperation, which is the boundary perception of authority and responsibility (Figure 3). The role of HoDs in different cooperation models is illustrated in the following sections.

## 6 Findings

### 6.1 Role of middle managers (HoDs) in Y University

#### 6.1.1 Delineating the requirements of staff responsibilities in hierarchy

**Figure 4:**
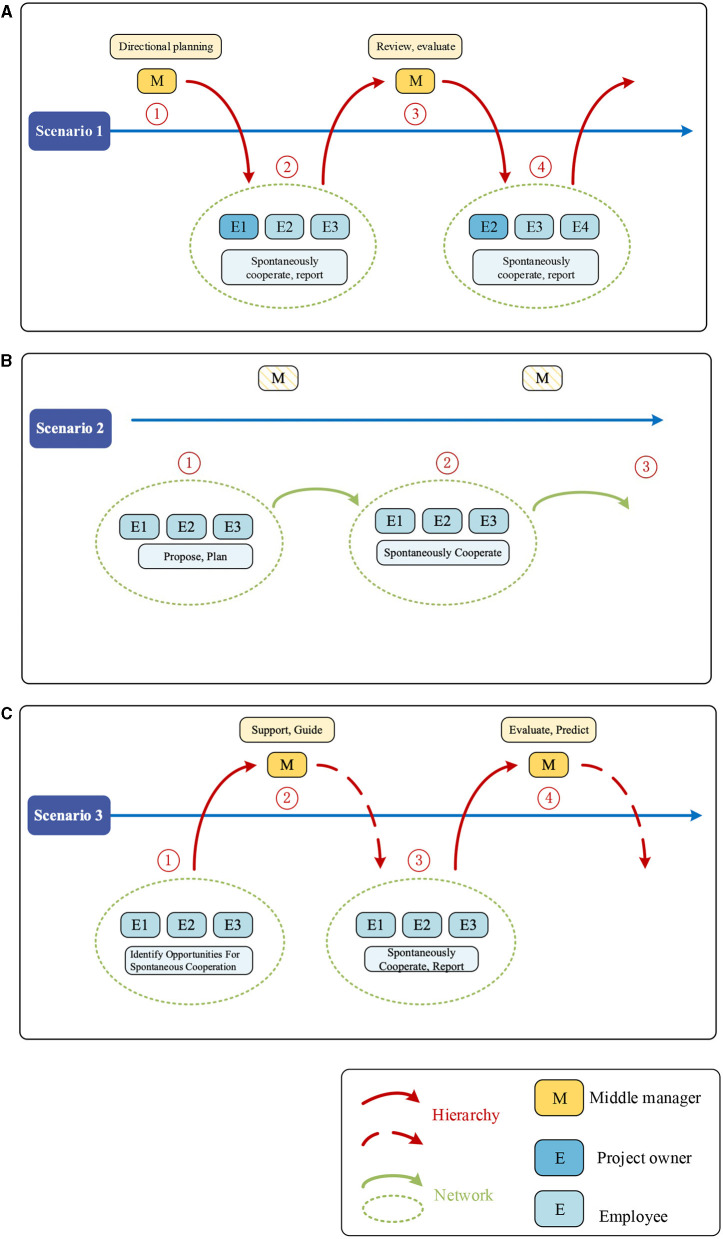
**(A–C)** Cooperation scenarios.

HoDs play a visible role in delineating staff responsibilities to ensure orderly cooperation. As shown in scenario 1 in Figure 4A, some interviewees mentioned that top-down collaboration from the middle level is common and based on duty requirements and job descriptions. HoD first initiated cooperation to make general arrangements for future development. For example, HoD uncovered opportunities during formal meetings or informal discussions and set up a direction for 1-year planning. Then, HoD pass on the general ideas to grassroots employees, who transfer the ideas into a detailed project, as explained by the two team leaders:

“*A previous project was started by the Dean and HoD in a chat. They both thought it was an excellent opportunity and then started the collaboration. It should be a general intent to work together to reach the top and then come back to do it precisely (P1)*.”

“*There will be a project owner in the team and helping to promote cooperation. Employees work together for 1 month then it's possible there will have some adjustment in the following stage. For example, we worked with department A in the first phase, and in the second phase, we needed to work with department B. The project will have new people coming in, but we need to follow the general arrangement from the middle level (P2)*.”

According to Figure 4A, E1, E2, and E3 (employees) involved in the project, cooperated based on duty, and followed orders from the superior. E1 worked as a project owner, but the three worked as a team on an equal footing. After approximately a month of cooperation, they reported periodic achievements to the HoD. HoD reviewed the progress report and suggested directions for the next stage. E1, E2, and E3 will continue to the next project, and there may be a change in team members. For example, E1 may require withdrawal from the second phase of project cooperation, and E2 may want to take charge as the project owner. Simultaneously, E4 wants to join in the project. The relatively flexible cooperation of employees in scenario 1 was based on authority and orders, and the division of tasks required the assignment and gatekeeping of superior. Employees are required to work in accordance with their responsibilities and to report to their superiors for review. Hence, the HoDs are the key controllers in scenario 1.

#### 6.1.2 Inspiring the sense of staff autonomy in network

HoDs provide invisible motivation within the organizational culture. This kind of invisible motivation is evident in the personal open-minded leadership of the HoD, the liberal organizational culture, and the kind atmosphere created by HoD. Moreover, HoD promotes equality and mutual respect within the department. Grassroots employees are encouraged to communicate and share ideas at any time. Employees' work results are recognized so that it further motivates them to be actively involved in work. This inspires employees' autonomous participation in innovation and cooperation. It can be said that HoDs tangibly and intangibly influence the development and process of grassroots cooperation.

As shown in scenario 2 in Figure 4B, the most apparent difference between scenarios 1 and 2 is that HoDs have no visible role in this cooperating process. Cooperation breaks through the logic of power and order, which promote the in Figure 4B, E1, E2, and E3 found some possible opportunities for cooperation based on their research backgrounds, research interests, etc. They proposed the cooperation spontaneously for articles or student engagement programs. One assistant professor and one HoD mentioned the following:

“*We need to research; everyone wants to publish articles and apply for funds. We work on academic papers and research funding due to similar research interests... Either for the future career development or research interest in interdisciplinary academic cooperation. We just did it when we wanted to… Our superior is a visionary leader and is open to encouraging various innovative collaborations. It doesn't matter as long as it doesn't interfere with the duty (P3)*.”

“*I just make sure all the daily work is on the right way. I will not intervene in any other things. Everyone is free to do just fine. We can say the atmosphere of our academy and the university is friendly and open. Personally, I'm not preferring to bind people with orders. Some interdisciplinary research are very popular nowadays. I also encourage staff to try. It's also helpful for student cultivation. So, it is good to hear that teachers are motivated to collaborate on research (P4)*.”

As shown in Figure 4B, there was no significant effect of the HoDs in the second cooperation scenario. Flexible cooperation among grassroots employees breaks through the hierarchy's logic of power and order. HoDs are more like atmosphere makers who inspire a sense of staff autonomy for innovation and cooperation.

#### 6.1.3 Translucent hand of explicit and implicit connections when hierarchy and network coexist

HoDs play an integrated role wherein hierarchy and network coexist in a knowledge organization. Based on empirical data, we found that HoDs play a varied role. It is a combination of delineating the requirements of staff responsibilities in the hierarchy and inspiring a sense of staff autonomy in a network. This integrated role is conceptualized as a translucent hand of explicit and implicit connections. While explicit connections under the hierarchy promote fixed cooperation based on duty regulations, implicit connections under the network are viewed as a flexible cooperation that is vision- and value-driven. Sometimes, they provide tangible guidance based on hierarchy, and sometimes create an invisible atmosphere based on the network. Hence, middle managers play separate roles in explicit and implicit connections.

As shown in scenario 3 in Figure 4C, the cooperation differs from that in scenario 1 in the initial stages. E1, E2, and E3 identified potential opportunities for cooperation in daily activities. Owing to shared recognition, the three cooperated spontaneously without the order of HoDs. It should be noted that this matters outside their duties. Nevertheless, they initiated cooperation because of a shared vision of value creation, such as career development and student cultivation. Grassroots staff proactively initiated cooperation and sought advice or support from middle-level when they achieved milestones. According to one assistant professor and one educational developer:

“*The employee's professional horizon is limited and may not have broad exposure to information. We need guidance from the HoDs sometimes. For example, what is not done correctly and needs to be adjusted. Some resources need to be called upon later (P5)*.”

“*One staff found me for the requirement analysis to develop the function; other teachers could also benefit from this project. But this is not in my performance appraisal. I want to help teachers while assisting in teaching and learning; it could also benefit student cultivation. Of course, I will talk to my Head when I report on my work, and if he says it's inappropriate, I'm sure I won't do it (P6)*.”

The reporting to the HoD mentioned here has two possibilities. First, grassroots staff cannot progress to the next stage because of their limited vision. Sustainable cooperation requires visual guidance. Thus, they would report to the HoD for evaluation, who will suggest whether they should pause or switch direction. Second, limited capacity and resources allow the grassroots to seek support from the middle level during the cooperation. Therefore, grassroots employees in scenario 3 have a certain degree of flexibility and freedom to cooperate and network, but the appropriate involvement and coordination of HoDs are still required when necessary. HoDs can be selectively involved in cooperation or provide a matchmaking platform.

In summary, middle managers are tangible hands in a traditional hierarchical organization because of their power and authority in explicit connections. However, middle managers have a transparent role in network organization because implicit connections break through the logic of power and promote network cooperation. When hierarchy and network coexist in an organization, it means that the visible (non-transparent) and invisible (transparent) roles come together to shape the hybrid role of HoDs within Y University, which is conceptualized as the translucent hand. In a knowledge-based organization such as Y University, the role of members is not static but dynamic and flexible depending on the situation.

### 6.2 The interfering factor of HoDs in adopting hierarchical or network cooperation in Y University

We found a hybrid organizational structure at Y University. The collected data revealed the operation mechanism of Y University, in which hierarchy and network can coexist (Table 2). Moreover, the relationship between hierarchy and network in Y University is not static but dynamic. Hierarchy and network occupy different positions in each of the three cooperation scenarios.

**Table 2:** Comparison and the interfering factor of three cooperation scenarios.

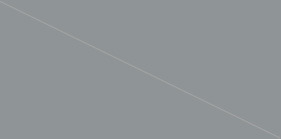	**Type of cooperation**	**Type of connection**	**Visibility of middle managers**	**Type of interaction**	**Boundary perception of authority and responsibility (interfering factor)**
Cooperation scenario 1	Hierarchical cooperation	Explicit connection	Visible	Rule-followed compulsory interaction	Strong
Cooperation scenario 2	Network cooperation	Implicit connection	Invisible	Value-created selective interaction	Unconscious
Cooperation scenario 3	Hierarchical and network cooperation	Explicit and implicit connections	A mix of visible and invisible	Rule-followed compulsory interaction and value-created selective interaction	Weak

Based on the above findings for the first research question, we further investigated the factors that impact HoD's choice while adopting hierarchical or network cooperation at Y University. We grounded two focused codes as rule-followed compulsory interaction and value-created selective interactions (see Figure 3). Specifically, rule-followed interaction is power- and order-driven, and compulsory interaction often occurs under a hierarchical structure. HoDs need to participate in this kind of interaction because of duties, as per their job descriptions. Moreover, value-created interaction is vision-driven, which is a selective interaction that occurs more in a network structure depending on the HoDs' personal consciousness. HoDs are flexible to participate in different cooperation to promote value creation based on a shared vision. Value creation refers to developing several interdisciplinary projects driven by a shared vision of student cultivation.

As shown in Table 2, the manifestation of hierarchy and network within Y University varied among the three cooperation scenarios. Hierarchical and network cooperation existed separately in scenarios 1 and 2, while coexisted in scenario 3. The underlying cause of this phenomenon is the presence of middle-level power involvement in the cooperation. Hence, based on the two focused codes, the theoretical code was developed as boundary perception of authority and responsibility to describe the factor that impacts the HoD's power involvement in hierarchical cooperation or network cooperation. In addition, the strength of the HoD's boundary perception of authority and responsibility determines the HoD's participation in different cooperation scenarios.

The HoD's personal delineation of boundaries of authority and responsibility determines the participation in the cooperation. HoDs are required to participate in and oversee tasks within their duties, such as school-level cooperative projects and university–enterprise collaboration. In this situation (cooperation scenario 1), the strength of the boundary perception of authority and responsibility was high because of the structural power of the hierarchy. One HoD mentioned:

“*Some school or university-level projects are significant to us. I need to know what our staff are doing and what they intend to do in the future. I am more by the rules. Otherwise, if the staff did something wrong, I don't know; they just came to me and asked for help when the situation got terrible and complicated. I will be very passive (P7)*.”

When hierarchical and network cooperation coexist (cooperation scenario 3), the strength of HoD's boundary perception of authority and responsibility is low. This is because the middle-level decides whether to participate through selective intervention. It can be said that the network cooperation needs some support and assistance from the middle level when it reaches a certain stage of development. The presence of hierarchy is less intense and tacitly allows for the existence of network. One senior associate professor and one HoD narrated:

“*Some projects are started by ourselves. Then we found the results looks good. We want to make the project bigger and benefit more teachers and students. This is the time need to report to the middle level, and the HoD will take the lead in some resources and network coordination. Our strength is relatively limited, and some resource mobilization needs the support of the middle level (P8)*.”

“*I've been working as HoD for some years now. Some HoDs will indeed be more severe, but I will not mandate what my employees must and cannot do. Just ensure the job is done well. I will not interfere with the rest. These are their own business… I usually have a coffee chat with our colleagues to find out how they are doing recently, see if they need any help, or just talk about their career planning (P9)*.”

There are some situations wherein HoDs do not consider themselves leaders of their employees. They haven't realized it is necessary to manage employees through responsibility or authority. HoD is only a job description in the organizational structure. Equality and mutual respect are the main subjects in the organizational atmosphere. Hence, HoDs will not interfere with the free cooperation of the grassroots. Grassroots cooperation is vision-driven and focuses on value creation, which is not forced by orders and is beyond the control of hierarchy. Hence, in cooperation scenario 2, we proposed that middle managers are unaware of the boundary perceptions of authority and responsibility. The middle level has no sense of power and is unwilling to interfere with the flexible and networked cooperation of the grassroots. One lecturer and one HoD noted:

“*Like lecturers, we work together on SURF projects because our research interest is similar, and the projects are not too difficult to apply for. Sometimes the student will come to me with a research idea, and a colleague has strong research skills in this field, so I will ask if she or he wants to join the project… For young teachers, we are happy to participate in the program, both from the perspective of enriching our teaching experience and helping student cultivation, and HoD won't stop us (P10)*.”

“*As HoD, I think I'm innovation-welcomed. I encourage our staff to expand their social circle and actively participate in various activities. And we welcome cross-collaboration within or outside the department. These can enlarge the knowledge and enrich the experience; it doesn't matter how much we do… we encourage staff to learn new things. In this enlightened atmosphere, our communications were smooth and efficient (P11)*.”

In summary, HoD involvement based on structural power determines hierarchical cooperation, whereas motivation and encouragement based on invisible climate creation enable network cooperation. Moreover, HoDs' boundary perception of authority and responsibility determines whether they should be involved in hierarchical or network cooperation.

## 7 Discussion

### 7.1 Extending the role of middle managers in an organizational context where hierarchy and network coexist

Our study makes theoretical contributions to the literature on network organizations and middle managers in the higher education context. First, this study revealed three existential relationships between hierarchy and network by identifying three cooperation scenarios. Unlike previous studies, we identified a new hybrid organizational structure in knowledge-intensive organization. Hence, we propose an integrated perspective on the role of middle managers in universities, where hierarchy and network coexist. The visible role of middle managers in the hierarchy and their invisible role in the network form their composite role. As per the empirical data, we extended and conceptualized the new role of middle managers as a “translucent hand of explicit and implicit connections” in a knowledge-intensive organization.

The single organizational structure of hierarchy or network can explain the role of middle managers in cooperation scenarios 1 and 2 but cannot interpret the composite role of middle managers in the new cooperation scenario 3, where hierarchy and network coexist. Previous studies have interpreted the role of middle managers in a single organizational structure differently. Middle managers in hierarchical organizations have been described in terms of their structural (Kallenberg, 2015) and functional effects (Anicich and Hirsh, 2017) on strategic management and operations. However, it is still being determined whether the position of middle managers in the network organizations can be strengthened (Balogun and Johnson, 2004) or weakened (Hellawell and Hancock, 2001). Additionally, the role of middle managers in the coexistence scenario of hierarchy and network remains uncovered.

Hence, this study develops a new description of middle managers as the translucent hand of explicit and implicit connections from an integrated perspective that hierarchy and network coexist in an organization. Middle managers' participation from structural power implies a hierarchical representation of the organizational structure. Contrarily, middle managers are akin to mood setters in the network that provides a covert impetus for corporate culture. The translucent hand helps interpret the composite role of middle managers, which tangibly and intangibly impact university cooperation simultaneously. In a complex and ever-changing environment, middle managers' role is not simple and static but complex and dynamic. The bridging position of the middle-level inevitably brings about a multifaceted role for middle managers in balancing different organizational layers. Hierarchy-based structural power and network-based freedom climate creation by middle managers jointly promote knowledge innovation in the digital intelligence age. Moreover, translucent hand interpretation has practical implications for university middle managers' career development and work balance.

### 7.2 Revealing the factor influencing middle managers' role choice

Social comparisons and organizational identification impact middle managers' performance feedback and strategic role (Tarakci et al., 2018). The ability to synthesize information impacts middle managers' role of filtering information flow (Shi et al., 2009; Kallenberg, 2015). However, these are studied in the context of a hierarchy-based single organizational structure. The factor that influences middle managers' role choices in a hybrid organizational structure (as shown in cooperation scenario 3) still remains uncovered.

Based on empirical data, two types of interactions between middle managers and grassroots employees were realized, providing insight into the different involvements of middle managers in hierarchy and network. Rule-following compulsory interaction refers to the supervision and evaluation of HoDs based on departmental job responsibilities in a hierarchical cooperation (as shown in cooperation scenario 1). Value-created selective interaction refers to the atmosphere created by HoDs that provides space for flexible network cooperation for employee knowledge creation (as shown in cooperation scenario 2).

However, cooperation scenario 3 revealed a hybrid organizational structure in which hierarchical and network cooperation coexisted. This involves differentiating how middle managers engage in hierarchical and network collaboration. Based on rule-followed compulsory interaction and value-created selective interaction, we consider the boundary perception of authority and responsibility as an important factor for distinguishing hierarchies and networks through power involvement in a knowledge organization. The idea of the boundary perception of authority and responsibility is introduced from the sociological literature, which originates from the social boundary theory proposed by Simmel (2007), who considered that a conflict might begin with a boundary that separates powers. Simmel (2007) stated, “Wherever the interests of two elements are concerned with the same object, the possibility of their coexistence depends on a boundary line within the object separating their spheres.” The social boundary perspective guided our distinction regarding the choice of middle managers in hierarchical or network cooperation.

The degree of boundary perception also reveals the degree of involvement from the HoD's hierarchical power. Strong boundary perception implies substantial control over the structural power of HoDs in hierarchical cooperation. Unconscious boundary perception indicates that HoDs have no clear boundaries for power and control, thus emphasizing equality and freedom for network cooperation. Additionally, a new scenario of coexistence of hierarchy and network is proposed in our study. The low boundary perception of HoDs provides scope for flexible network cooperation with selective intervention from the hierarchy.

## 8 Conclusion

“Collective intelligence, the quantity and quality of intellectual collaboration, is well-managed freedom” (Serrat, 2017). Network organizations provide a flexible platform for collective intelligence that promotes accessible and adaptive organizational development. In this study, we adopt an integrated view and put forward the coexistence of hierarchy and network. The relationship between hierarchy and network in Y University is dynamic since hierarchy and network occupy different positions in each of the three cooperation scenarios. We proposed that network can break through the logic of power, while does not mean eliminating it. Both hierarchy and network are essential for universities in the ever-changing environment.

Organizational members are indispensable components of organizational operations (Sathiyaseelan, 2023). The role of middle managers at Y University is not static, but dynamic in the information age. Building on previous studies of HoDs that examined functional and structural roles, we further refined the roles in terms of power involvement as the translucent hand of explicit and implicit connections from an integrated perspective of hierarchy and network. The HoD's boundary perception of authority and responsibility determines the awareness of involvement in different cooperation. The findings of this study have practical implications for SFCUs' organizational structure changes and propose a hybrid hierarchy-network organizational structure for Y University. However, we conducted the exploration in one SFCU. A comparison of multi-universities from different continents could be considered to investigate the characteristics of network organizations and cooperation mechanisms in different university systems.

## Data availability statement

The original contributions presented in the study are included in the article/supplementary material, further inquiries can be directed to the corresponding author.

## Ethics statement

The studies involving humans were approved by Research Ethics Committee of XJTLU. The studies were conducted in accordance with the local legislation and institutional requirements. The participants provided their written informed consent to participate in this study.

## Author contributions

JL: Conceptualization, Data curation, Writing – original draft. XZ: Conceptualization, Supervision, Writing – review & editing.

## Appendix

**Table A1:** Interview information.

**Participant no**.	**Department**	**Position**	**Type of interview**	**Times of interview**
P1	Institute of Leadership and Education Advanced Development	Team Leader	Face to Face	1
P2	Institute of Leadership and Education Advanced Development	Team Leader	Face to Face	1
P3	Department of Health and Environmental Sciences	Assistant Professor	Face to Face	1
P4	Learning Institute for Future Excellence	HoD	Face to Face	2
P5	Department of Economics	Assistant Professor	Online	1
P6	Educational Development Unit	Educational Developer	Face to Face	1
P7	Institute of Leadership and Education Advanced Development	HoD	Online	1
P8	Department of Health and Environmental Sciences	Senior Associate Professor	Face to Face	1
P9	Department of Health and Environmental Sciences	HoD	Face to Face	1
P10	Department of Mechatronics and Robotics	Lecturer	Face to Face	2
P11	Department of Biological Sciences	HoD	Face to Face	1
